# Roles of Brand Benefits and Relationship Commitment in Consumers’ Social Media Behavior around Sustainable Fashion

**DOI:** 10.3390/bs13050386

**Published:** 2023-05-06

**Authors:** Tae Rang Choi, Jisoo Ahn

**Affiliations:** 1 Department of Strategic Communication, Texas Christian University, Fort Worth, TX 76109, USA; 2 Department of Communication and Media, Dong-Eui University, Busan 47340, Republic of Korea

**Keywords:** sustainability, benefits, brand relationship, commitment, behavioral intention, social media

## Abstract

As climate change continues, environmental sustainability has become a popular topic among brands and consumer groups. The fashion industry has detrimental impacts on the natural environment; however, little is known about how brand benefits can help sustainable fashion brands develop relationships with consumers and promote consumer behavior. This study focuses on Instagram to investigate how consumers’ perceived brand benefits predict relationship commitment, electronic word-of-mouth (eWOM), and purchase intention. Prior studies have overlooked the possible effects of various benefits. This study outlines five benefits of sustainable fashion brands: inner self-expression, social self-expression, warm glow, green, and economic benefits. Results from a survey of sustainable fashion brand followers on Instagram showed that eWOM positively related with economic benefits and negatively with warm glow and green benefits. Findings further indicated a mediating effect of relationship commitment between benefits and consumers’ behavior. Lastly, the level of environmental attitude influenced the mediating impact of relationship commitment. The implications of these findings are discussed, and suggestions for future research are provided.

## 1. Introduction

Ralph Lauren hit the headlines during the Tokyo 2020 Olympics when the company unveiled its sustainably produced U.S. Olympic and Paralympic Team USA Closing Ceremony Parade Uniforms and apparel collection, featuring a sustainable high-tech cotton dyeing and manufacturing process. This innovation exemplifies fashion brands’ endeavors to defy the fashion industry’s reputation for environmental damage. Indeed, fashion production accounts for 10% of the world’s carbon emissions, produces toxic waste, dries up water sources, and pollutes rivers and streams [1,2]. Clothes also become 9.2 million tons of waste annually, yet global consumer spending in this category is projected to continually rise, reaching $2.9 billion in 2025 [3]. The fashion industry’s detrimental effects have compelled a growing number of global fashion consumers to commit to sustainability and to urge fashion brands to be environmentally responsible [4]. Granskog et al. further noted that consumers have recently begun to support sustainable fashion brands and to make purchase decisions based on brands’ related initiatives, such as using sustainable materials, recycling, and shipping with eco-friendly packaging [4].

Social media wields great influence over individuals’ brand-related activities based on benefits in general; however, little is known about, in the context of sustainable fashion brands, what brand benefits associated with sociopsychological and economic angles would drive promoting relational commitment with such brands and one’s positive behaviors on social media toward the brands. Doing such research can fertilize the literature by finding out how social media facilitates a driving force of consumers’ environmental consumption behavior [5]. Because of these reasons, brands and organizations are leveraging these platforms to connect with prospective consumers and champion their sustainability efforts by offering varied benefits [4,5]. One example is Earthday.org using social media to promote “Make Every Day, Earth Day” on every year’s Earth Day. The organization’s efforts are intended to propel its campaigns and pique consumers’ interest in sustainability by providing goods of participation, ideally resulting in building commitment to the organization and, thus, inspiring people to behave sustainably. As these online venues offer consumers an array of benefits, social media can enrich brands’ performance and relationships with consumers [6].

Despite a growing emphasis on sustainability in the fashion industry, limited research has investigated how sustainable fashion brands can encourage desired consumer behavior on social media [7]. Scholars have called for research on factors that influence sustainable fashion consumption and bolster consumer-brand relationships in a digital context [1,8,9]. Drawing on social exchange theory (SET) [10], this study focuses on benefits of fashion brands that claim sustainability as a crucial aspect. Customers are thought to have acknowledged the advantages of eco-friendly products as a reason to purchase such items [11]. Our work fills a gap in the literature by examining consumers’ perceived benefits of sustainable fashion brands on Instagram. Specifically, the following benefits are considered: inner self-expression, social self-expression, warm glow, green, and economic benefits. We further investigate the mediating role of relationship commitment in the association with brand benefits and consumers’ eWOM behavior and purchase intentions. Two research questions are posed to explore the moderating role of environmental attitudes in cultivating this relationship, given that such attitudes represent a key moderator of green purchases [12,13]. Not all apparel consumers are environmentally oriented. This research hence also addresses how consumer groups’ responses (as evidenced by environmental attitudes) vary toward brand benefits. Findings contribute to the theoretical advancement of sustainable marketing by providing a richer understanding of brand benefits and the role of relationship commitment in consumers’ behavioral intentions. Besides being of theoretical interest, our results offer meaningful managerial insight on variables affecting brands’ Instagram followers’ actions.

### 1.1. Sustainable Fashion Brands on Instagram

Sustainability refers to “development that meets the needs of the present without compromising the ability of future generations to meet their own needs” [14] (p. 54). This concept includes three pillars: environmental, social, and economic. Increasing climate change and environmental issues have led a number of consumers to voice concerns about environmental sustainability. A recent consumer research report indicated that about 60% of consumers are willing to alter their consumption habits to alleviate environmental consequences [15]. This report further suggested that, in terms of purchasing goods, more than 70% of consumers look for brands that hold sustainable values (e.g., offering clean products, exhibiting environmental responsibility, and supporting recycling). Sustainability has thus become an impetus behind consumers’ purchase decisions [15,16]. In light of this consumer movement, the present study focuses on environmental sustainability, particularly in the fashion industry.

A cresting green wave among consumers and fashion’s deleterious impacts on the environment have pushed the fashion industry to take a stand on being sustainable, birthing the notion of sustainable fashion. Sustainable fashion is both a movement and “a process to change and foster the fashion products and also to ensure the fashion and ecological integrity of these products” [1] (p. 199). Conscious fashion brands create clothes that respect the environment by using organic raw materials, recycling materials (e.g., plastic bottles), avoiding harmful bleaches, and other eco-conscious actions. Patagonia, an outdoor clothing brand, is an exemplar of sustainable fashion: the company uses recycled plastics and polyester to produce new polyester fibers for their clothing. Amid this sustainable movement, social media offers an appealing avenue where consumers can benefit from brands’ presence and develop associated relationships [17]. Social media platforms enable brands to showcase specific attributes while reaching sustainable (and potential) consumers, given that consumers engage with social media based on topics in which they are interested [5]. The positive effects of media and social interaction on consumers’ environmental behavior therefore position social media as an ideal communication setting [18].

Our work focuses on Instagram, a visually oriented platform with more than 500 million active daily users. Instagram is an appropriate choice because it is consumers’ most preferred social channel for “following” brands [19]. Fashion is an inherently aesthetic industry. As of February 2021, Instagram excelled in gaining fashion-related traffic; this social media channel boasted the greatest number of posts and the highest engagement rates among social media outlets [20]. Taking Instagram as a sample platform offers rich insight into how consumers respond to the various benefits that environmentally sustainable fashion brands provide through this channel.

### 1.2. Social Exchange Theory

Grounded in economics, psychology, and sociology, SET is a useful theoretical framework to fathom how the complexity of social constructs is relevant to human behaviors [21]. Fundamentally, SET postulates that people interact and take actions to boost their benefits while diminishing their costs [10], meaning that SET proposes that exchange arises from mutually beneficial interactions between parties based on subjective cost–benefit analysis. For example, individuals tend to expect various benefits, such as economic return and emotional and relational benefits, when they behave in a certain way [22,23]. This notion has been applied to consumer behavior studies. Consumer research involving SET shows that consumers are willing to build relationships with brands when the advantages of doing so exceed the costs [22,24]. A number of studies have also demonstrated that consumers seek various returns and enjoy such advantages related to self-identity, social status, and hedonia from brand interaction, generating stronger relationship commitment and behavioral intentions [25,26,27]. To illustrate, people engage in brand-related activities when they anticipate receiving benefits from the brand; exchanges will be terminated once costs surpass benefits. In particular, sustainability alone does not drive consumer behavior [28,29]. Drawn from SET, it remains necessary to understand, which benefits motivate brand relationship exchanges in conveying to consumers that sustainable fashion consumption will be rewarding [27,30]. Moreover, the application of SET has been deemed fruitful in social media research [21]. Hence, it is suitable and reasonable to employ SET as a theoretical framework in this research.

Because SET views exchange as social behavior, the concept of “exchange” in this theory encompasses economic costs and benefits (e.g., money, time) along with social costs and benefits (e.g., respect, love, friendship, self-gratification). In this light, SET maintains that numerous benefits influence consumers’ behavior and promote supportive partnerships. Previous literature found that consumers displayed word-of-mouth (WOM) intentions upon perceiving hedonic and economic benefits from a bike-sharing app [23]. Social benefits also apparently influence consumers’ behavior on social media [28], and perceived benefits play significant roles in consumers’ purchase intentions [30,31].

One possible challenge is that people weigh actions’ benefits and costs because environmentally friendly consumption provides value (e.g., protecting the environment) that comes at a cost (e.g., increased effort) [32]. With respect to sustainable fashion, Lundblad and Davies’ qualitative study revealed that people were inspired to buy green clothing as a way of showcasing their values, expressing themselves, feeling confident and happy, and engaging in environmentally responsible consumption [33]. Social media, as it relates to general fashion and hedonic consumption, also affords consumers diverse benefits in flaunting their personalities and lifestyles, elevating their social status, and connecting with fashion brands that meet their needs [8,34]. SET in this context suggests that sustainable fashion brands might offer assorted benefits (by virtue of being eco-friendly) when using Instagram to communicate with consumers. Perceived benefits can lead to positive brand relationships and consumer behavior [23]. Although these are reasonable predictions aligned with SET, less is known about the consequences of certain benefits on brand relationships and consumer behavior with regard to sustainable fashion brands on social media, particularly Instagram.

### 1.3. Benefits of Sustainable Brands

In the realm of consumer—brand relationships, benefits reflect “the personal value consumers attach to the brand attributes, that is, what consumers think the brand can do for them” [35] (p. 411). Consumers are inclined to demonstrate a stronger commitment to greener brands and to engage in environmentally sustainable behavior when faced with possible benefits, such as environmental, monetary, and psychological advantages [31,32,34,36,37,38]. Early studies underlined the impacts of green benefits on consumers’ behavior. For example, Hartmann and Apaolaza-Ibáñez identified positive influences of environmental benefits on consumers’ intentions to purchase a green energy brand [36]. Scholars have also observed significant roles of psychological, immaterial benefits (e.g., self-expression, positive feelings) in determining consumers’ motives around earth-friendly behavior and relational commitment [32,37,39]. Yet, personal evaluations of sustainable brands’ benefits are largely paramount [37,40].

Most work in this stream has either framed perceived benefits as a single variable or focused on a particular type [30,31,41]; that is, how different benefits might affect consumers’ sustainable behavior remains unclear. While little is known about how brand-related benefits on social media inform consumers’ behavior, such advantages can have diverse impacts on Instagram fashion brands’ enactment of sustainability—consumers anticipate tangible and intangible benefits from environmentally friendly brands [34]. Consumers are also pivotal in inspiring sustainable fashion consumption, as their spending on fashion is projected to continue to rise [42]. Today’s consumers are deeply interested in environmental sustainability and aim to spread the word [43]. It is accordingly crucial to delineate the brand benefits that drive consumers to engage in environmentally sustainable behavior and how these benefits support brand relationships [44,45]. Therefore, this study proposes five benefits that consumers can gain from sustainable fashion brands on Instagram. These assets are expected to serve as motivations behind eWOM and purchase intention, complementing prior research.

#### 1.3.1. Inner Self-Expression Benefits

Brands’ symbolic functions have been well documented and capture how brands and products can facilitate the expression of one’s self-concept [46]. In the current context, inner self-expression is defined as a consumer’s perceptions of the extent to which a brand mirrors the type of person they are (i.e., one’s inner self) [25]. Research on fashion consumption has taken clothing as a means of self-identity formation and self-expression [30]. For instance, some may wear trendy clothes to display a “chic” self, whereas others may don clothes made of recycled plastic bottles to project a “green” self-image. Lundblad and Davies interviewed regular consumers of sustainable fashion and discovered that consumers purchase sustainable clothing because this type of apparel enables them to project their self-image, values, and personality (i.e., to express who they are) [33]. Research has also revealed that consumers exhibit greater intentions to spread positive feedback about brands that support them in expressing their inner selves [47]. Social media offers an arena where individuals can construct and present their self-concept by showing which brands they favor and how they “do good” [48,49]. It is therefore logical to assume that consumers may be willing to engage in eWOM and make purchases when they expect to benefit from expressing their inner selves through the sustainable fashion brands they follow on Instagram. Stated formally:

**H1a:**  *Inner self-expression benefits will be positively associated with eWOM.*

**H2a:**  *Inner self-expression benefits will be positively associated with purchase intention.*

#### 1.3.2. Social Self-Expression Benefits

In addition to the inner self, the social self is another dimension of self-concept associated with a brand’s symbolic benefits [25,47,50]. Social self-expression embodies a consumer’s perceptions of the extent to which brands can foster one’s social self-image and help one make positive impressions on others [12,25]. Given that one’s possessions can become an extension of their self-identity, scholars have suggested that consumers can fulfill their needs in the social context by patronizing specific brands [51]. People might purchase from certain brands to gain social approval, present themselves in a positive light, or communicate a reference group identity [52,53,54]. In the same vein, Carroll and Ahuvia found that brands promoting social self-expressiveness led consumers to purchase from the brand and spread positive WOM for the brand [25].

Signaling theory asserts that people intend to behave altruistically to signal prosocial traits and present socially desirable selves [36]. Evidence has indicated that consumers are more likely to purchase green brands and to act sustainably when socially visible [26]. This tendency may arise from positive social perceptions of sustainability [32,37]. This inclination is especially relevant in the fashion context, as consumers often use fashion items to communicate their social selves. Khare showed that consumers’ desire to make a favorable impression and earn approval from a social group influenced their perceived benefits of earth-friendly apparel and led to purchase behavior [30]. Sustainable fashion brands should thus cater to consumers’ needs for social self-expression [30,55]. As people begin to behave more sustainably in public settings [32,36], scholars have come to identify the impact of social media on environmental sustainability [18]. Instagram, with its dynamic interactivity among users and high publicity, can foster social visibility and enable users to share certain aspects of themselves with others through earth-friendly brands [48,50]. We therefore expect consumers to engage with sustainable brands on Instagram given a belief that these brands can help them express their social selves. The following hypotheses are proposed accordingly:

**H1b:**  *Social self-expression benefits will be positively associated with eWOM.*

**H2b:**  *Social self-expression benefits will be positively associated with purchase intention.*

#### 1.3.3. Warm Glow Benefits

Warm glow is an internal emotional reward that denotes a “feeling of wellbeing as a consequence of the moral satisfaction engendered by contributing to the environmental common good” [36] (p. 1255). This concept is rooted in the belief that positive emotions (e.g., happiness, pleasure, joy) follow from doing good [56]. As such, earth-friendly behaviors could offer individuals emotional benefits and self-satisfaction. Behaving in a “green” manner has been shown to cause people to feel good and literally warm, as evidenced by a higher body temperature [57].

Warm glow has recently received growing attention in sustainable consumer research [32,56]. Empirical evidence points to a positive relationship between warm glow and purchase intention; that is, people appear likely to engage in green-branded energy consumption when motivated by warm glow benefits [36]. Similarly, consumers are more apt to purchase green brands—even for a premium price—because doing so leads them to feel better about themselves [58]. For example, using green airlines can cause customers to feel optimistic about helping the environment, resulting in favorable WOM [59]. Along the same lines, feeling happy and experiencing less guilt may drive consumers to purchase sustainable clothing. The warm glow concept suggests that people do good for altruistic and non-altruistic reasons. Given that egoistic motivations are tied to fashion consumption [33], consumers may experience a vicarious warm glow by purchasing sustainable clothing while fulfilling personal desires. As personal benefits influence eWOM [47], it is expected that internal, emotional benefits will inform consumers’ intentions to share positive WOM about such brands online. The following hypotheses are thus put forth:

**H1c:**  *Warm glow benefits will be positively associated with eWOM.*

**H2c:**  *Warm glow benefits will be positively associated with purchase intention.*

#### 1.3.4. Green Benefits

Green benefits are integral to environmentally friendly brands. These benefits involve consumers’ perceptions of the extent to which a brand is concerned about the environment, produces eco-friendly products, and can help protect the environment [37]. Prior research revealed that green benefits positively influence consumers’ behavioral intentions. For instance, the environmental benefits of a green energy brand have been shown to boost consumers’ purchase intentions [36]. Furthermore, consumers express intentions to continue their relationship with a brand and to purchase its products when the brand is thought to offer environmental benefits, such as having sustainable functionality, striving to reduce environmental issues, and providing greater environmental value than other brands [37,60]. A recent study [61] indicated that green benefits associated with remanufactured consumer goods positively affected the purchase intentions of consumers from the United Kingdom and China. Brands’ earth-friendly benefits are expected to play similar roles in consumers’ behavior related to sustainable apparel. Lundblad and Davies conducted in-depth interviews with current consumers of such apparel and observed that consumers seek out and purchase sustainable fashion because they wish to support sustainable manufacturing (e.g., using natural materials, plant-friendly production techniques, and recycled materials) [33]. This course of action allows consumers to contribute to a healthier environment, hence the following hypotheses:

**H1d:**  *Green benefits will be positively associated with eWOM.*

**H2d:**  *Green benefits will be positively associated with purchase intention.*

#### 1.3.5. Economic Benefits

Economic benefits have been suggested as a core motive in consumer and social media research; people seek economic rewards from brand relationships [62]. These benefits encompass the economic advantages that consumers can gain from a brand, including in terms of money (e.g., coupons, special deals, sale information), time (e.g., prompt customer service), and opportunity (e.g., giveaways) [63,64]. Social media is a prominent an effective place in which to present economic benefits. Economic rewards facilitate consumer–brand relationships and can encourage consumers to engage in brand-related activities (e.g., eWOM) on social media [48]. Social media brand research has shown that economic benefits (e.g., discount codes) generate more frequent consumer interaction and stronger intentions to continue a relationship with a brand and, by extension, purchase its products or services [17,64,65,66]. The same pattern is thought to hold for consumers of sustainable fashion brands on Instagram. Green products are generally believed to be more expensive than non-green products [26]. Price therefore represents a barrier to purchasing sustainable fashion [33]. Hence, people who follow sustainable fashion brands on social media may be likely to spread positive feedback about these brands and purchase apparel when receiving economic rewards. Brands’ economic benefits have garnered research attention; however, little work has concerned sustainable fashion. The following hypotheses are proposed thusly:

**H1e:**  *Economic benefits will be positively associated with eWOM.*

**H2e:**  *Economic benefits will be positively associated with purchase intention.*

### 1.4. Relationship Commitment

In consumer research, relationship theory argues that consumers can cultivate relationships with brands, and commitment is a tenet of successful long-term brand relationships [22]. Relationship commitment is defined in this context as consumers’ desire to maintain their relationship with a brand and their willingness to dedicate themselves to sustaining this valued relationship [67]. Committed consumers seek to keep this relationship and do right by the brand to acquire possible benefits [68,69]. The relational viewpoint holds relationship commitment as an influencing construct that leads consumers to strengthen their relationship with a brand now and in the future [70].

The literature on consumer–brand relationships suggests that benefits and commitment are central to behavioral intention during exchanges between brands and consumers, as consumers seek payback from their purchases [22,70]. The perceived benefits of a brand boost consumers’ desire to strengthen their relational bond with that brand [24]. Empirical findings from a range of domains demonstrate that various benefits contribute to relationship commitment [37,38,67]. In terms of social media brands, consumers are more likely to commit when they expect to receive advantages such as social, self-related, utilitarian, and hedonic benefits [38,40]. Economic benefits are positively associated with consumers’ brand commitment and likelihood of participating in brand-related activities [38,65]. Zhao et al. identified crowdfunding backers’ perceived benefits as a significant antecedent of relationship commitment; backers were more willing to continue their relationship with a cause when crowdfunding projects were seen as beneficial [24]. Brands pursue relationship enhancement by offering consumers diverse benefits [71]. Research on green brands [31,37] has reported that perceived benefits can mold long-term consumer–brand relationships. The aforementioned five benefits could then serve as motivators of consumers’ relationship commitment to sustainable fashion brands on Instagram as theorized:

**H3:**  *(a) Inner self-expression benefits, (b) social self-expression benefits, (c) warm glow benefits, (d) green benefits, and (e) economic benefits will be positively associated with relationship commitment.*

Relationship commitment plays a major part in consumer exchange and appears critical to securing valuable brand outcomes [70,72,73]. Consumers who are committed to a particular brand are willing to maintain the relationship and tend to partake in associated marketing outcomes, such as eWOM and actual purchases [67,71,74]. It is also found in the realm of sustainable marketing that when consumers sense that a relationship is beneficial, they do more sustainable behaviors and continue engaging in doing so [32,45].

Palmatier et al.’s meta-analysis of relationship marketing studies found the importance of commitment as a relational mediator, affected by relational benefits that consumers receive from their exchange partner (i.e., a brand) [75]. This finding has been supported by other individual studies in different areas. For example, commitment in the relationship mediated the relationship between backers’ perceived benefits and behavioral intentions to take part in crowdfunding projects [24]. In sustainable fashion context, greater perceived benefits sparked stronger commitment and greater purchase intention for both familiar and unfamiliar sustainable fashion brands [31].

Based on the relationships between benefits, commitment, and behavioral intentions (eWOM and purchase) including the mediating role of relationship commitment in different areas of marketing, we can assume that as consumers anticipate benefiting from brand relationships more, they may express stronger commitment to such relationships, and, in turn, are more likely to act on their eWOM and purchase intentions. Therefore, the study hypothesizes the following:

**H4:**  *Relationship commitment will mediate the relationship between (a) inner self-expression benefits, (b) social self-expression benefits, (c) warm glow benefits, (d) green benefits, and (e) economic benefits and eWOM.*

**H5:**  *Relationship commitment will mediate the relationship between (a) inner self-expression benefits, (b) social self-expression benefits, (c) warm glow benefits, (d) green benefits, and (e) economic benefits and purchase intention.*

### 1.5. Environmental Attitudes

While brands expect good images from consumers by providing various benefits, those benefits may not bring out the same results to consumers; that is, individual characteristics can make consumers perceive the brand’s marketing strategies differently and make different outcomes. For example, consumers’ orientation toward the environment can be linked to eco-friendly brands’ strategies and purchase behavior. Especially, different from general fashion consumption, which is influenced by hedonic motives, environmental attitudes can be important in sustainable fashion consumption. In fact, A considerable body of research in environmental psychology has underscored these attitudes as pivotal to one’s environmental orientation related to cognitive, affective, and behavioral commitment and intentions [12].

This present study defines environmental attitudes as a person’s “psychological tendency expressed by evaluating the natural environment with some degree of favour or disfavour” [76] (p. 80) and examines how this individual characteristic impacts on the relationship between benefits, relationship commitment, and eWOM and purchase intention mentioned above. Previous literature focused on the effects of environmental attitudes on behaviors: consumers with favorable environmental attitudes are more likely to purchase products and services from environmentally sustainable brands [12,13] and advocate for earth-friendly brands [77]. However, in order to establish targeted marketing strategies based on specific consumer characteristics, it is necessary to investigate how environmental attitudes affect the perception of a brand’s strategies (i.e., perceived benefits) and expected outcomes (i.e., relationship commitment, eWOM, and purchase intention). Since the related research is rare, it would be better to have research questions (RQs) rather than setting directions of the relationships to see the moderating role of environmental attitudes.

**RQ1:**  *How will environmental attitudes affect the relationships between consumers’ perceived benefits, eWOM, and purchase intentions, predicted in H1 and H2?*

**RQ2:**  *How will environmental attitudes affect the indirect effects of consumers’ perceived benefits on eWOM and purchase intentions through relationship commitment, predicted in H4 and H5?*

**Figure 1:**
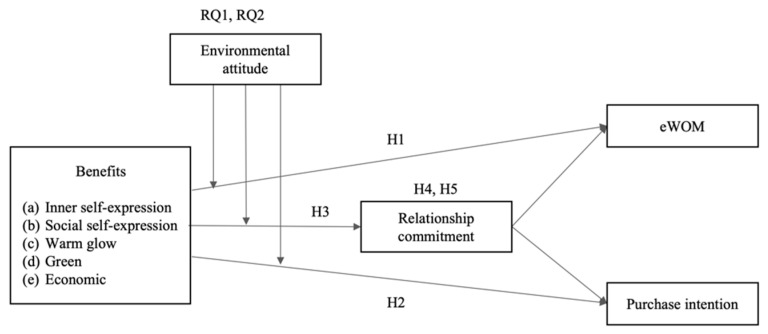
Proposed Model.

Our proposed research model is depicted in Figure 1.

## 2. Materials and Methods

### 2.1. Respondents and Procedure

An online survey was conducted among current Instagram users recruited from Amazon Mechanical Turk because this survey platform is confirmed as an effective way of high-quality data by recruiting the targeted participants [78]. Respondents were asked to sign a consent form and to complete a survey questionnaire. After respondents consented to participate, three steps were taken to ensure that respondents were eligible to take part in this study. First, those who were not current Instagram users were redirected to the end of the survey. They were then asked a second screening question to gauge the frequency of their Instagram usage; those who answered that they checked Instagram either “almost never” or “less than once a month” were again brought to the end of the survey. Lastly, respondents who stated that they did not currently follow any sustainable fashion brands on Instagram were led to the end of the survey. Respondents who met the qualifications were next asked to write down the name of a sustainable fashion brand they followed. They were then asked to answer a series of questions about their specified brand. Respondents were instructed to complete the main measures in the study first, followed by Instagram usage–related questions and demographic items. Respondents were finally debriefed and thanked for their participation.

Our initial sample contained 515 respondents. Those who failed to cite an existing sustainable fashion brand were excluded from subsequent analysis. Identified brands were cross-checked with the presence of the brand’s Instagram account. Additionally, respondents whose questionnaires were incomplete or which contained extreme or abnormally consistent rating patterns were removed. The final sample included 491 respondents. Among them, 51.3% were males and 47.9% were females; 0.8% preferred not to disclose their gender. Their average age was 34.3, ranging from 18 to 70 years. Most respondents designated their race as Caucasian (69.9%), followed by African American (15.1%) and Asian (11%). Regarding education, most respondents held a bachelor’s degree (58.5%) followed by a master’s degree (17.3%); a smaller proportion had no degree but had completed some college (9.4%). The majority of respondents were either single/never married (29.9%) or married (58.9%) and had children (57%).

More than half of respondents (61%) reported having used Instagram for more than 3 years. Respondents stated that they checked Instagram frequently, with roughly 80% visiting the platform either daily or several times a day. Nearly 37% posted to their Instagram feed or Instagram Story a few times per week; almost 22% did so either at least once a day or more than once a day. Respondents’ average usage duration was approximately 56 min per day, and they had about 723 followers on average. Complete sample characteristics for Instagram usage patterns are listed in Table 1.

### 2.2. Measures

All items were rated on a 7-point Likert scale (1 = strongly disagree, 7 = strongly agree) unless otherwise indicated. The reliability of all measures exceeded the desired threshold of 0.80.

#### 2.2.1. Benefits

To assess inner self-expression benefits, respondents were asked to rate to what extent the sustainable fashion brand they followed on Instagram helped them represent the type of person they were and their personality. Six items were drawn from prior work [48] with slight modifications to fit the context of this study (Cronbach’s α = 0.91). Five items evaluating social self-expression benefits were adapted from earlier research [12,54] and tailored to this research setting. These items were intended to measure the extent to which consumers believed they could express a positive social self-image and gain social approval/prestige through earth-friendly apparel brands (Cronbach’s α = 0.87). Warm glow benefits were measured based on five items from past studies [36,37]. These items concerned the extent to which consumers perceived the brand as helping them feel good about not harming the environment or creating a healthier earth (Cronbach’s α = 0.84). Four items on green benefits were employed to examine consumers’ perceptions that a brand, which appears concerned about the environment, is eco-friendly and presents environmental attributes relevant to respecting the environment and promoting environmental protection [37,60] (Cronbach’s α = 0.84). We focused on sustainable fashion brands that use Instagram when measuring economic benefits. After requesting that respondents think about sustainable fashion brands they followed on Instagram, they were asked to rate items showing how strongly they believed they gained monetary (e.g., coupons) and other economic advantages (e.g., time) from those brands. Three items were adapted from prior work [63,64] along with one self-created item (Cronbach’s α = 0.80). All scale items for each construct were averaged for analysis.

#### 2.2.2. Relationship Commitment

Three items from De Wulf et al. were employed to assess the extent to which consumers wanted to continue a relationship with the brand they followed on Instagram and their willingness to devote effort to maintaining that relationship [67] (Cronbach’s α = 0.84). Relevant items were revised slightly to align with the context of this study. A single index was created by averaging the item scores.

#### 2.2.3. Environmental Attitude

To understand individuals’ psychological tendencies to take action to save the environment, five items were adopted from Biswas and Roy [12] (Cronbach’s α = 0.85). All responses were averaged to create a single index score.

#### 2.2.4. eWOM

Six items evaluating the frequency of positive eWOM were adapted from Chow and Shi [79] and modified to reflect the features of Instagram (e.g., regramming). Respondents were asked to think about the brand they had specified at the beginning of the survey and to indicate how often they engaged in eWOM-related activities for that brand (Cronbach’s α = 0.92). These items were measured on a 7-point scale ranging from 1 (never) to 7 (almost always), and item scores were averaged to produce an index score.

#### 2.2.5. Purchase Intention

The extent to which consumers were willing to buy goods from their identified brand was evaluated using three items from Erkan and Evans [80] (Cronbach’s α = 0.80). An index score was generated by averaging the item scores.

#### 2.2.6. Fashion Interest

Three items assessing fashion interest were adapted from Fu and Kim [81]. These items were attempted to uncover individuals’ interest in fashion and their engagement with general fashion products (Cronbach’s α = 0.90). Similar to prior measures, the item scores were averaged to generate an index score.

More details regarding all measures and descriptive statistics are reported in Appendix A.

### 2.3. Data Analysis

Hierarchical ordinary least squares regression was used to examine H1a–e, H2a–e, and H3a–e, which addressed relationships between five types of benefits and consumers’ relationship commitment, eWOM, and purchase intentions. We controlled the impacts of respondents’ posting frequency, the period of using Instagram, gender, parent status (i.e., with or without children), environmental attitudes, and fashion interest in our analysis. The controls were entered first, and key variables (i.e., benefits) were entered in the next steps to assess the increase in *R*^2^ and effects of variables [82]. Multicollinearity was checked based on tolerance values and variance inflation factors.

We used the PROCESS macro to analyze mediating effects (H4 and H5) and moderated mediating effects (RQ1 and RQ2). Specifically, we investigated the indirect effects of each benefit on eWOM and purchase intention through relationship commitment via PROCESS Model 4; conditional indirect effects by environmental attitude were analyzed with Model 8. The bootstrapping analysis involved 5000 samples.

## 3. Results

### 3.1. Effects of Benefits

We hypothesized (H1a–e) that all benefits would be positively related to eWOM. As shown in Table 2, only H1e (economic benefits; *β* = 0.28, *p* < 0.001) was supported. Warm glow (*β* = −0.10, *p* < 0.05) and green (*β* = −0.11, *p* < 0.05) benefits were negatively associated with eWOM, and no significant associations were found with inner and social self-expression benefits.

H2a–e theorized positive relationships between benefits and purchase intention. Results indicated that warm glow (H2c; *β* = 0.14, *p* < 0.01) and green (H2d; *β* = 0.17, *p* < 0.01) benefits were significantly related to purchase intention; however, no significant relationships were observed for inner and social self-expression and economic benefits.

H3a–e predicted that the five types of benefits would be positively associated with relational commitment. Table 2 reveals that relationship commitment is related positively with inner self-expression (*β* = 0.16, *p* < 0.001), social self-expression (*β* = 0.23, *p* < 0.001), warm glow (*β* = 0.09, *p* < 0.05), and economic (*β* = 0.23, *p* < 0.001) benefits but related negatively with green benefits (*β* = −0.12, *p* < 0.01). As such, H3 was partially supported.

### 3.2. Indirect Effects of Benefits

We proposed a mediating role of relationship commitment in the impacts of benefits on eWOM (H4a–e) and purchase intention (PI; H5a–e). The indirect effects of inner self-expression (B_eWOM_ = 0.24, 95% CI = [0.160, 0.333]; B_PI_ = 0.10, 95% CI = [0.052, 0.148]), social self-expression (B_eWOM_ = 0.31, 95% CI = [0.214, 0.410]; B_PI_ = 0.10, 95% CI = [0.052, 0.160]), warm glow (B_eWOM_ = 0.21, 95% CI = [0.113, 0.310]; B_PI_ = 0.07, 95% CI = [0.036, 0.121]), and economic (B_eWOM_ = 0.15, 95% CI = [0.092, 0.205]; B_PI_ = 0.10, 95% CI = [0.060, 0.137]) benefits on eWOM and purchase intention through relationship commitment were significant (Table 3). As individuals thought that the sustainable fashion brands they followed on Instagram could help them convey their self-identity, make a positive social impression, experience positive feelings about being involved in the brand’s good aims, and receive information about economic rewards, they tended to maintain their relationship with the brand. They subsequently recommended the brand to others and wanted to purchase its products; however, green benefits did not play a significant mediating role on either eWOM or purchase intention. Thus, H4 and H5 were partially supported.

### 3.3. Conditional Effects of Benefits

RQ1 entailed whether environmental attitudes would moderate the direct effects of benefits on eWOM and purchase intention. Among the interaction terms, warm glow × environmental attitudes and green benefits × environmental attitudes had negative effects on the direct association of the respective benefits with eWOM; the economic benefits × environmental attitudes pair demonstrated a positive impact. Even though all groups displayed significant effects, those with lower environmental attitudes (e.g., negative attitudes toward environmental issues) elicited stronger effects (see Table 4). For purchase intention, all interaction terms except for economic benefit × environmental attitudes exerted significant and positive effects on the direct association (see Table 5). The effects were also more pronounced for respondents with weaker environmental attitudes.

Regarding RQ2, the moderated mediation results revealed a significant moderating role of environmental attitudes in the indirect effects of warm glow (B_eWOM_ = 0.07, 95% CI = [0.029, 0.133]; B_PI_ = 0.03, 95% CI = [0.010, 0.052]) and green (B_eWOM_ = 0.07, 95% CI = [0.029, 0.140]; B_PI_ = 0.03, 95% CI = [0.011, 0.064]) benefits on eWOM and purchase intention through relationship commitment. Specifically, for warm glow benefits, all attitude groups were significant for eWOM but showed differential effects (low: B = 0.16, 95% CI = [0.064, 0.263]; medium: B = 0.22, 95% CI = [0.130, 0.331]; high: B = 0.29, 95% CI = [0.177, 0.416]). We therefore observed stronger mediating effects among respondents with stronger environmental attitudes. Similar patterns emerged for the conditional indirect effect of warm glow benefits on purchase intention. In the case of green benefits, the mediating effects on eWOM and purchase intention were significant for respondents with moderate (B_eWOM_ = 0.11, 95% CI = [0.024, 0.202]; B_PI_ = 0.05, 95% CI = [0.011, 0.093]) and strong (B_eWOM_ = 0.17, 95% CI = [0.067, 0.303]; B_PI_ = 0.08, 95% CI = [0.028, 0.139]) environmental attitudes. Apart from these two benefits, the mediated relationships between other benefits and both eWOM and purchase intention were not moderated by environmental attitudes. See Table 6 for a summary of the findings.

## 4. Discussion and Conclusions

Embracing environmental sustainability, which signals how brands care for the planet, has become common in the fashion industry due to consumers’ growing attention to sustainability. Studies on environmentally friendly brands have documented how perceived benefits influence people’s environmentally sustainable behavior [32,37,38]. However, most work has explored benefits in general, with little empirical light cast on the links between brand benefits, relationship constructs, and behavioral outcomes. To address these deficiencies, the present research scrutinized the effects of five brand benefits—inner self-expression, social self-expression, warm glow, green, and economic benefits—on consumers’ eWOM and purchase intentions toward sustainable fashion brands on Instagram. This research also sought to examine the roles of relationship commitment and environmental attitudes between these benefits and behaviors.

The results explicate how self-expression benefits can predict consumers’ positive relationship commitment to sustainable fashion brands. The stronger consumers’ beliefs that they can reveal different aspects of themselves, the deeper and more enduring their relationships with sustainable brands. These findings echo research showing that people use social media to express their different selves [83]. As social connection lies at the heart of social media, earth-friendly brands may offer an ideal way to showcase a positive side of the self and to promote a socially acceptable self [49]. Economic benefits led consumers to participate in positive eWOM and was likely to foster long-term brand relationships. Requesting that consumers tag friends and share brands’ posts are prevalent social media marketing strategies; consumers may engage more in eWOM behavior for the chance to earn rewards. These findings substantiate work in relationship marketing suggesting the positive effects of economic benefits on relational outcomes and eWOM [38,64]. Our results also reinforce the SET perspective [10] in that different benefits appear to be significant predictors for sustainable fashion brands on Instagram.

Interestingly, warm glow and green benefits were negative drivers of eWOM behavior. Because social relationship factors influence eWOM on social media, consumers may not engage in brand information exchange or brand advocacy when benefits seem irrelevant to social interaction; eWOM is voluntary [84]. Within the interactive social media environment, people can share positive or negative feedback on others’ posts [85]; consumers with internal benefits may be unwilling to share their opinions and instead maintain their own reasons for doing good inside. These consumers possibly will not spread the word about brands because the benefits they desire are not expected to be returned by doing so. Another explanation involves personal inclinations such as green advertising skepticism and perceived information utility; consumers who are skeptical of the benefits of green marketing will not find the advertised information especially useful [86]. Compared with consumers who seek sustainable products for external advantages, individuals pursuing environmental and internal benefits might be more likely to doubt the messaging in a sustainable brand’s Instagram posts [87]. This skepticism may hinder eWOM. Subsequent studies could present a content analysis of sustainable fashion brand followers’ Instagram posts to further explore our unanticipated outcomes. Results would extend the understanding of sustainable fashion brand consumers’ behavior.

The findings of the present research also expand the relationship marketing and sustainable brand literature [32,67,75] by unearthing a significant mediating role of relationship commitment in the associations between all benefits (except green benefits) and consumer behavior. Consumers, for whom these brands offer advantages in projecting a personal self-image, presenting a positive social self-image, experiencing heartfelt warmth, and gaining economic benefits, are more likely to extend their relationships with brands. These consumers will then be more apt to share positive feedback about brands and to purchase brands’ sustainable items. These benefits can in turn position eWOM and purchase likelihood as functions of consumers’ relationship commitment towards sustainable fashion brands followed on Instagram. The identified mediating effects of relationship commitment are akin to those in earlier studies [24,71]. The current results add to empirical evidence in support of SET [10,24], confirming that consumers seek varied benefits from sustainable brands to fulfill different needs and tend to behave in ways that will produce desired outcomes. Moreover, these findings resonate with consumer–brand relationship research suggesting that consumers’ perceived benefits are conducive to relationship commitment and stronger brand relationships [22,37,70].

The moderating effects of environmental attitudes further contribute to the knowledge of environmentally sustainable brands. Among benefits, warm glow, green (negative relationship), and economic (positive relationship) benefits have significant relationships with eWOM, and these relationships were stronger for those who expressed less favorable attitudes toward the environment. On the other hand, consumers with less favorable environmental attitudes showed a little more purchase intention when they perceived all types of benefits except economic benefits. This finding buttresses the premise of the value-belief-norm theory of environmentalism [88] and rational choice theory [89,90] that people’s internalized attitudes toward the natural environment can enhance intentional environmental actions (i.e., via eWOM and purchase intention). In addition, we found the relevant types of benefits which can be affected eWOM and intention and be differed by individuals’ intrinsic motives regarding the environment.

The finding of the indirect relationship considering benefits, relationship commitment, and eWOM/purchase intention is more noteworthy in that the moderating effect of environmental attitudes is significant for a warm glow and green benefits, and higher attitudes brought out the stronger degree of mediation. To be specific, when comparing the moderated mediation and mediation results, people overall were not likely to commit to a relationship with the brand and eWOM or purchase the brand’s product when thinking about green benefits, whereas people with favorable environmental attitudes had positive outcomes in this relationship. Perhaps, consumers possessing positive environmental attitudes might choose to cultivate relationships with brands whose missions align with their interests (i.e., protecting the environment), sparking eWOM and product/service purchases [88]. Our findings enrich the knowledge base on sustainable fashion brands by suggesting that although fashion is relevant to hedonic consumption, consumers’ favorable environmental attitudes lead to desirable branding outcomes when brands’ green benefits are highlighted.

### 4.1. Theoretical Implications

Several key findings of this research contribute to understanding consumers’ psychology toward marketing and consumer–brand relationship strategies of sustainable brands. First, we expanded the existing literature on sustainable brands, particularly in the fashion context, with a multiangle perspective. While prior research has extensively examined brand benefits as a single variable or focusing on a particular benefit, scant attention has been paid to the influences of various benefits and to the context of environmentally friendly brands [31]. However, the present study tested the sociopsychological, emotional, and economic benefits of sustainable fashion brands on social media and showed the importance of having other brand benefits as well as earth-friendly benefits to enhance brand relationships and positive behavioral consequences. Second, our mediation results support that relationship commitment remains a crucial factor in building relationships with sustainable fashion brands on social media [22,72]. This not only adds to the body of research about the mediating effect of relationship commitment in consumer—brand relationships but also applies the SET to the context of sustainable brands. Lastly, by examining the moderating effects of environmental attitudes, we could compare the direct and indirect effects of various benefits on eWOM and purchase intention and find the differences between those who were more and less favorable attitudes toward the environment. These findings can suggest future research to consider individual characteristics, which are relevant to the topic of study, to concretely interpret the general results by groups.

### 4.2. Managerial Implications

The findings of the present research also yield valuable managerial implications for marketers of sustainable fashion brands and their media strategies. Brands’ success on social media is contingent on fulfilling consumers’ needs [6], and this holds true for sustainable fashion brands in terms of offering consumers benefits. It is crucial to leverage various benefits to encourage positive eWOM and sustainable fashion purchases on Instagram. To boost followers’ relationship commitment, brand managers need to develop social media strategies that elevate followers’ perceptions of sustainable fashion brands as personally beneficial. Providing an array of benefits and satisfying followers will facilitate robust brand relationships. When offering assorted benefits, marketers should note that sustainable brands must exhibit transparency and sincerity to create intended marketing outcomes [91]. Fast fashion brands’ (e.g., H&M) common sustainability efforts (e.g., placing recycling bins in stores) seldom appeal to today’s smart consumers and can ultimately have negative environmental impacts. The move toward sustainability is anticipated to persist in the fashion industry. Brand-related benefits, when presented with genuine commitment, are essential in helping firms rise above the competition to build and maintain healthy brand relationships. These connections can help brand managers realize desired advertising and social media marketing outcomes.

### 4.3. Limitations and Suggestions for Future Studies

Despite its revelations and contribution, this research is not without limitations. Respondents followed sustainable fashion brands on Instagram, but because we did not institute age parameters, our sample may not capture generational views on environmental issues. Considering that Generation Z appears most interested in sustainability and purchasing from sustainable brands [92], this generation may be unique in terms of consumers’ perceived brand benefits and consumer–sustainable fashion brand relationships. Future research could conduct cross-sectional studies to provide additional insights regarding similarities and differences across generations (e.g., Generation Z, Baby Boomers) in sustainable fashion consumption patterns and behavioral engagement with sustainable fashion brands on Instagram [16].

Second, the focus of this research was on Instagram. Given that consumers and brands continue to find information and interact via emerging channels, such as voice artificial intelligence devices and metaverse platforms [93], newer media channels could be examined to discern how sustainable fashion brand benefits influence consumers’ relationship commitment and behavior.

Lastly, some measurement issues should be pointed out. Since the PROCESS macro we employed analyzed the relationships between benefits × environmental attitude and eWOM/relationship commitment/purchase intention separately, measurement errors might be induced. Partial least squares structural equation modeling could instead be applied for hypothesis testing while controlling for error. In that case, a simpler model would be recommended to create interaction terms (e.g., by specifying a moderating route). Another issue is about common method variance. Our survey included all independent and dependent variables, and the data were collected from a single source. This can promote a higher tendency of marking the variables in the same manner (i.e., systematic variance) and a positive correlation between the variables. The topic of the survey was linked with social desirability (e.g., sustainable behaviors for nature), this error might have appeared more frequently. To reduce or control the common method variance in future research, it is recommended to measure independent and dependent variables from different sources [94].

These limitations notwithstanding, our research bolsters the literature on environmental sustainability and sustainable brands, particularly in the fashion industry. Findings shed additional light on the underlying mechanism by which sustainable fashion brands’ brand-related benefits contribute to consumer–brand relationships. This work also presents actionable managerial implications to guide sustainable brands in shaping consumer behavior.

## Figures and Tables

**Table 1:** Descriptive Statistics for Instagram Usage Pattern.

	M (SD) or %
Usage period	
Less than 6 months	3.5
7–12 months	7.5
1–2 years	27.9
3–5 years	36.9
More than 5 years	24.2
Visit frequency	
A few times a month	6.5
A few times a week	13.0
Daily or almost daily	34.6
Several times a day	45.8
Posting frequency	
Never	1.4
Rarely	7.5
A few times a year	8.8
A few times a month	22.6
A few times a week	37.3
Once a day	13.6
More than once a day	8.8
Usage duration per day (minutes)	56.52 (115.81)

**Table 2:** OLS Regression Predicting Relationship Commitment, eWOM, and Purchase Intention.

		Relationship Commitment		eWOM		Purchase Intention	
		β	t	β	t	β	t
Step 1	Frequency of Posting	0.07	2.38 *	0.13	4.50 ***	0.02	0.68
	Usage Period	−0.05	−1.86	−0.10	−3.22 **	0.01	0.46
	Gender	−0.05	−1.77	−0.04	−1.53	−0.01	−0.43
	Marital Status	0.01	0.35	0.02	0.60	0.09	2.42 *
	Children	−0.04	−0.99	−0.11	−2.90 **	0.02	0.40
	Fashion Interest	0.16	4.61 ***	0.24	6.97 ***	0.12	3.18 **
	Environmental Attitude	0.24	6.18 ***	−0.01	−0.31	0.18	4.11 ***
Step 2	Inner Self-expression	0.16	3.64 ***	0.06	1.29	0.02	0.37
	Social Self-expression	0.23	4.81 ***	−0.02	−0.45	0.09	1.62
	Warm Glow	0.09	1.99 *	−0.10	−2.14 *	0.14	2.79 **
	Green	−0.12	−2.70 **	−0.11	−2.51 *	0.17	3.47 **
	Economic	0.23	6.81 ***	0.28	7.88 ***	−0.02	−0.45
Step 3	Relationship Commitment			0.37	8.07 ***	0.27	5.55 ***
		Adj *R*^2^ = 0.647 △*R*^2^ = 0.155 *** F(12,478) = 72.92 ***		Adj *R*^2^ = 0.638 △*R*^2^ = 0.048 *** F(13,477) = 67.54		Adj *R*^2^ = 0.588 △*R*^2^ = 0.026 *** F(14,476) = 51.03 ***	

Note. * *p* < 0.05, ** *p* < 0.01, *** *p* < 0.001; All values indicate standardized *β* value.

**Table 3:** Indirect Effects of Benefits on eWOM and Purchase Intention through Relationship Commitment.

Benefits	eWOM	Purchase Intention
Inner Self-expression	0.24 [0.160, 0.333]	0.10 [0.052, 0.148]
Social Self-expression	0.31 [0.214, 0.410]	0.10 [0.052, 0.160]
Warm Glow	0.21 [0.113, 0.310]	0.07 [0.036, 0.121]
Green	0.07 [−0.017, 0.156]	0.03 [−0.007, 0.067]
Economic	0.15 [0.092, 0.205]	0.10 [0.060, 0.137]

**Table 4:** Conditional Effects of Benefits on eWOM by Environmental Attitude.

	Benefits				
	Inner Self-Expression	Social Self-Expression	Warm Glow	Green	Economic
Conditional Direct Effects					
EA Low	−0.04 [−0.169, 0.096]	−0.08 [−0.228, 0.061]	−0.29 [−0.430, −0.144]	−0.27 [−0.408, −0.137]	0.37 [0.259, 0.490]
EA Medium	0.03 [−0.076, 0.144]	−0.06 [−0.187, 0.068]	−0.23 [−0.374, −0.095]	−0.26 [−0.400, −0.114]	0.35 [0.267, 0.442]
EA High	0.10 [−0.023, 0.233]	−0.04 [−0.182, 0.112]	−0.18 [−0.354, −0.011]	−0.24 [−0.424, −0.059]	0.33 [0.229, 0.438]
Conditional Indirect Effects					
EA Low	0.21 [0.132, 0.296]	0.27 [0.169, 0.384]	0.16 [0.064, 0.263]	0.04 [−0.038, 0.129]	0.15 [0.093, 0.230]
EA Medium	0.23 [0.155, 0.327]	0.30 [0.210, 0.407]	0.22 [0.130, 0.331]	0.11 [0.024, 0.202]	0.15 [0.093, 0.206]
EA High	0.26 [0.162, 0.378]	0.34 [0.240, 0.449]	0.29 [0.177, 0.416]	0.17 [0.067, 0.303]	0.14 [0.082, 0.199]
Index of Moderated Mediation	0.03 [−0.015, 0.082]	0.04 [−0.007, 0.080]	0.07 [0.029, 0.133]	0.07 [0.029, 0.140]	−0.01 [−0.048, 0.022]

**Table 5:** Conditional Effects of Benefits on Purchase Intention by Environmental Attitude.

	Benefits				
	Inner Self-Expression	Social Self-Expression	Warm Glow	Green	Economic
Conditional Direct Effects					
EA Low	0.15 [0.061, 0.237]	0.23 [0.138, 0.326]	0.30 [0.207, 0.393]	0.31 [0.217, 0.393]	0.002 [−0.081, 0.084]
EA Medium	0.13 [0.058, 0.203]	0.21 [0.128, 0.294]	0.29 [0.200, 0.382]	0.29 [0.199, 0.384]	−0.003 [−0.065, 0.060]
EA High	0.11 [0.027, 0.197]	0.19 [0.094, 0.285]	0.28 [0.171, 0.294]	0.28 [0.160, 0.397]	−0.01 [−0.081, 0.068]
Conditional Indirect Effects					
EA Low	0.08 [0.043, 0.137]	0.09 [0.044, 0.147]	0.06 [0.022, 0.105]	0.02 [−0.018, 0.057]	0.10 [0.061, 0.152]
EA Medium	0.10 [0.051, 0.150]	0.10 [0.051, 0.159]	0.08 [0.042, 0.132]	0.05 [0.011, 0.093]	0.10 [0.062, 0.138]
EA High	0.11 [0.055, 0.172]	0.11 [0.055, 0.178]	0.11 [0.056, 0.167]	0.08 [0.028, 0.139]	0.09 [0.054, 0.136]
Index of Moderated Mediation	0.01 [−0.006, 0.035]	0.01 [−0.003, 0.031]	0.03 [0.010, 0.052]	0.03 [0.011, 0.064]	−0.01 [−0.031, 0.015]

**Table 6:** Summary of Findings.

Hypotheses and Research Questions	Support/Key Findings
H1: (a) Inner self-expression benefits, (b) social self-expression benefits, (c) warm glow benefits, (d) green benefits, and (e) economic benefits → eWOM (+)	H1e: supported
H2: (a) Inner self-expression benefits, (b) social self-expression benefits, (c) warm glow benefits, (d) green benefits, and (e) economic benefits → purchase intention (+)	H2c and H2d: supported
H3: (a) Inner self-expression benefits, (b) social self-expression benefits, (c) warm glow benefits, (d) green benefits, and (e) economic benefits → relationship commitment (+)	H3a, H3b, H3c, and H3e: supported H3d: green benefits → relationship commitment (-)
H4: (a) Inner self-expression benefits, (b) social self-expression benefits, (c) warm glow benefits, (d) green benefits, and (e) economic benefits → relationship commitment → eWOM	H4a, H4b, H4c, and H4e: supported
H5: (a) Inner self-expression benefits, (b) social self-expression benefits, (c) warm glow benefits, (d) green benefits, and (e) economic benefits → relationship commitment → purchase intention	H5a, H5b, H5c, and H5e: supported
RQ1: How will environmental attitudes affect the relationships between consumers’ perceived benefits, eWOM, and purchase intentions, predicted in H1 and H2?	Environmental attitudes had negative (positive) effects on the direct associations between warm glow and green (economic) benefits with eWOM Environmental attitudes had positive effects on the direct associations between all benefits, except economic, with purchase intention
RQ2: How will environmental attitudes affect the indirect effects of consumers’ perceived benefits on eWOM and purchase intentions through relationship commitment, predicted in H4 and H5?	Environmental attitudes moderated the indirect effects of warm glow and green benefits on eWOM and purchase intention through relationship commitment

## Data Availability

The data of this study are available upon reasonable request.

## Appendix A

**Table A1:** Descriptive Statistics for Measurement Items.

		M	SD
Inner self-expression benefit		5.39	1.03
Brand X * allows other people to understand who I am		5.30	1.21
Brand X helps me represent what kind of person I am		5.48	1.18
Brand X helps me disclose who I am to the world		5.30	1.25
Brand X can craft my identity		5.26	1.36
Brand X lets me express myself		5.55	1.18
Brand X lets me shape my own identity/personality		5.46	1.24
Social self-expression benefit		5.55	0.96
Brand X helps me make a positive impression on other people		5.52	1.17
Brand X has a positive impact on what others think of me		5.45	1.24
Brand X helps me feel socially acceptable		5.51	1.20
I like to be seen wearing Brand X		5.64	1.13
I enjoy it when people know I am wearing Brand X		5.64	1.16
Warm glow benefit		5.76	0.86
I can feel better because I do not harm the environment with Brand X		5.69	1.12
I can feel good because I help to protect the environment with Brand X		5.80	1..10
I have the feeling of contributing to the well-being of humanity and nature with Brand X		5.79	1.06
I feel pleased and happiness with Brand X		5.75	1.08
I feel inspired by Brand X		5.76	1.13
Green benefit		5.92	0.85
Brand X is environmentally friendly		5.98	1.02
Brand X has more environmental benefits than other apparel brands		5.77	1.08
Brand X helps to prevent environmental issues		5.90	1.03
Brand X respects the environment		6.03	0.96
Economic benefit “On Instagram, Brand X …”		4.90	1.17
helps me to get coupons, discounts, bonuses, or special deals		4.88	1.40
helps me to get sales information early		5.07	1.40
helps me to participate in lotteries		4.47	1.78
helps me to get fast responses when I have a question about brand, product, or service		5.18	1.31
Relationship commitment		5.28	1.08
I am willing to go the extra mile to remain a customer of Brand X		5.21	1.21
I feel loyal towards Brand X		5.38	1.18
Even if Brand X would be more difficult to buy, I would still keep buying it		5.26	1.30
eWOM behavior “Thinking about Brand X, how often do you do the following activity?”		4.57	1.46
Tagging people in a comment on Brand X’s Instagram postings.		4.27	1.86
Regramming Brand X’s posting on my Instagram.		4.31	1.84
Posting Brand X-related photo, video, text, etc. on my own Instagram.		4.22	1.84
Recommending Brand X to others.		4.83	1.51
Saying positive words about Brand X to others.		5.03	1.49
Introducing the Brand X’s Instagram to others.		4.74	1.71
Purchase intention “When thinking about Brand X’s overall activities on Instagram …”		5.60	0.94
It is very likely that I will buy Brand X’s product		5.64	1.06
I will purchase Brand X’s product next time I need a product		5.56	1.13
I will definitely try Brand X’s new product		5.59	1.13
Environmental attitude		5.65	0.91
It is important to me that the products I use don’t harm the environment		5.60	1.16
I often consider the potential environmental impact of my actions when making many of my consumption decisions		5.63	1.14
I am concerned about wasting the resources of our planet		5.73	1.19
I would describe myself as environmentally responsible		5.68	1.10
I am willing to be inconvenienced in order to take environmentally sustainable actions		5.63	1.17
Fashion interest		5.30	1.27
Fashion clothing means a lot to me		5.22	1.40
I am very interested in fashion clothing		5.38	1.35
I am very much involved in/with fashion clothing		5.30	1.42

Note. * Brand X replaced to the brand name the respondent specified.
